# Respiratory viruses in the pre and post‐pandemic periods in an Italian tertiary hospital

**DOI:** 10.1002/iid3.909

**Published:** 2023-08-15

**Authors:** Flavio De Maio, Barbara Fiori, Delia M. Bianco, Maurizio Sanguinetti, Michela Sali

**Affiliations:** ^1^ Dipartimento di Scienze di Laboratorio e Infettivologiche Fondazione Policlinico Universitario “A. Gemelli”, IRCCS Rome Italy; ^2^ Dipartimento di Scienze biotecnologiche di base, Cliniche intensivologiche e perioperatorie—Sezione di Microbiologia Università Cattolica del Sacro Cuore Rome Italy

## Abstract

We have appreciated the article published by Bardsley and colleagues describing the seasonal circulation of respiratory syncytial virus (RSV) in UK children, and we hope to contribute to increase information on this intriguing and elusive topic. We describe our epidemiological trend with the aim to add a small brick to the current knowledge regarding respiratory infections due to RSV and other respiratory viruses in an era that is changing due to a radical change in the evaluation of respiratory symptoms following the pandemic event.

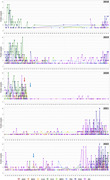

Despite having similar symptoms, human respiratory viruses differ in genomic features, seasonality and transmission mode.^1^ The use of non‐pharmaceutical interventions (NPIs), such as social distancing or face‐masking, to reduce Coronavirus disease 19 (COVID‐19), dramatically affected respiratory viruses spreading patterns.^2^ Furthermore, establishment and improvement of national and global surveillance systems and the increased public awareness of the health burden of respiratory viruses lead to a renewed interest in exploring their epidemiology.

As surveillance programmes were operating for respiratory syncytial virus (RSV) and influenza viruses in the prepandemic period, they might be representative examples of how NPIs impacted on global respiratory virus circulation. Indeed, RSV represents the most important cause of infant hospitalization in high‐income countries, causing mainly bronchiolitis whose treatment remains difficult and elusive.^3^

NPIs and behavioral changes massively influenced respiratory virus transmission; the end of lockdown periods corresponded to a higher incidence of RSV in paediatric populations and in immunocompromised individuals.^4^ This is probably related to the fact that RSV is often transmitted through close contact in childcare facilities and schools. Indeed, in 2021 RSV incidence increased, after being remarkably low in 2020.^5^

Moreover, some experts have raised concerns about a potential RSV epidemic in the future due to so‐called “immunity debt” in the population.^6^, ^7^ Similarly, the circulation of influenza viruses, which appeared significantly reduced during the COVID‐19 pandemic in different countries, increased with the withdrawal of NPIs.^8^

In this brief commentary, we report the trends observed from 2018 to 2022 in our clinical practice at Fondazione Policlinico Universitario Agostino Gemelli in Rome, a tertiary hospital contributing to national and regional surveillance programmes. We hope to contribute to the broadening knowledge regarding infections due to respiratory viruses in the postpandemic landscape, in which there have been radical changes in how we evaluate respiratory symptoms and illnesses.^9^, ^10^ All the analyzed nasopharyngeal and throat swabs were collected from people presenting to the emergency department with respiratory symptoms.

Increased global awareness resulted in an increased number of tests for respiratory viruses: counts doubled from 2018 to 2019 and remained steady in the following years.

As depicted in Figure 1, in prepandemic years (2018–2019) respiratory swab positivity rate at our hospital remained comparable, with relative values of 5.8% and 6.6%. This accounted for 267/4585 and 733/11080 positive tests, respectively. The number of emergency department visits remained relatively stable in the 2 years, 82.076 in 2018 and 81.826 in 2019. Thus, 4 and 10 people per 1000 emergency department attendees tested positive for respiratory viruses in 2018 and 2019, respectively. The number of tested samples doubled, which could be due to increased availability of multiplex testing assays and may represent the beginning of a change in the diagnostic assessment of respiratory infections.

**Figure 1:**
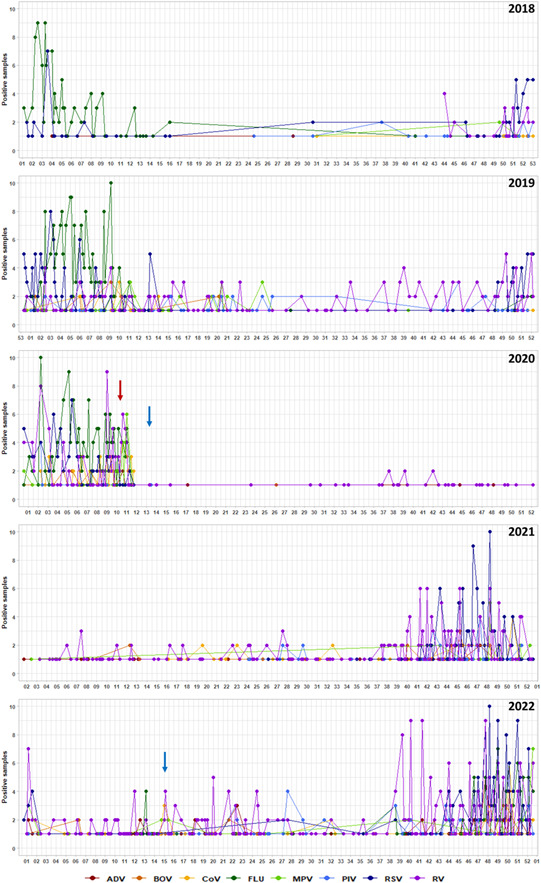
Schematic representation of the respiratory virus trend between 2018 and 2022. Respiratory viruses, molecularly detected, are described grouped for year in the line charts. Red line shows the first detection of SARS‐CoV‐2 in Italy, while the blue lines evidence the period with severe/moderate application of NPIs in Italy. RSV trend is showed: samples investigated for RSV, between 2018 and 2022, in adult and paediatric populations are assessed as negative or positive. ADV, adenovirus; BOV, bocavirus; CoV, human coronavirus (excluded SARS‐CoV‐2), FLU, influenza virus; MPT, human metapneumovirus; PIV, parainfluenza virus; RSV, respiratory syncytial virus; RV, rhinovirus.

After the emergence of the pandemic (2020), despite an increased number of tests, the total positivity rate decreased to 2.1% (531/25862), before going up to 4.9% (556/11340) in 2021 and to 5.4% (884/16232) in 2022. The decrease in the total positivity rate in 2020 corresponds to sharp fall in emergency departments attendances (57.895). The number of emergency department visits remained low in 2021 at 64.069.

Until 2020, influenza was the most prevalent respiratory virus, with a peak from January to March. Conversely, influenza circulation significantly dropped in the postpandemic period. We observed a positivity rate of 4.7% on 2686 analyzed samples in 2018, 4.4% on 4909 samples in 2019 and 3.5% on 4964 samples in 2020. In 2021, positivity rate reached a low of 0.1% of 1909 samples and climbed to 4.6% of a total of 2497 samples in 2022. These data are in line with previous findings that describe respiratory virus epidemiology in other countries.

In prepandemic years, small peaks of RSV and rhinovirus (RV) were reported in the winter months, whereas a seasonality change was depicted in the period 2021–2022 when peaks of RSV and RV were detected in the autumn.

Samples tested for RSV had a positivity rate of 14.1% of 504 analyzed samples in 2018, falling to 11.1% of 1415 samples in 2019 and 2.7% of 2887 samples in 2020. The rate then climbed up again to 10.9% of 1293 samples in 2021 and 9.6% of 1924 samples in the following year.

While Bardsley and colleagues reported an increase in RSV incidence in paediatric patients, we observed a different trend in our population. We stratified the paediatric population in infants‐toddlers (aged 0–2 years), preschoolers (aged 3–5 years), middle childhood people (group 1: aged 6–8 and group 2: 9–11 years), young teens (aged 12–15), teenagers (aged 16–18).

Interestingly, positivity rate to RSV in paediatric patients was meaningfully different in preschoolers in 2021, that shows a relative value of 23.4%, while in prepandemic period (2018–2019) accounted 5.6% and 8.9%; 6.3% in 2020 and in the final tail of pandemic 8.2% (Figure 2A). No noteworthy change was observed in the other paediatric subgroups.

**Figure 2:**
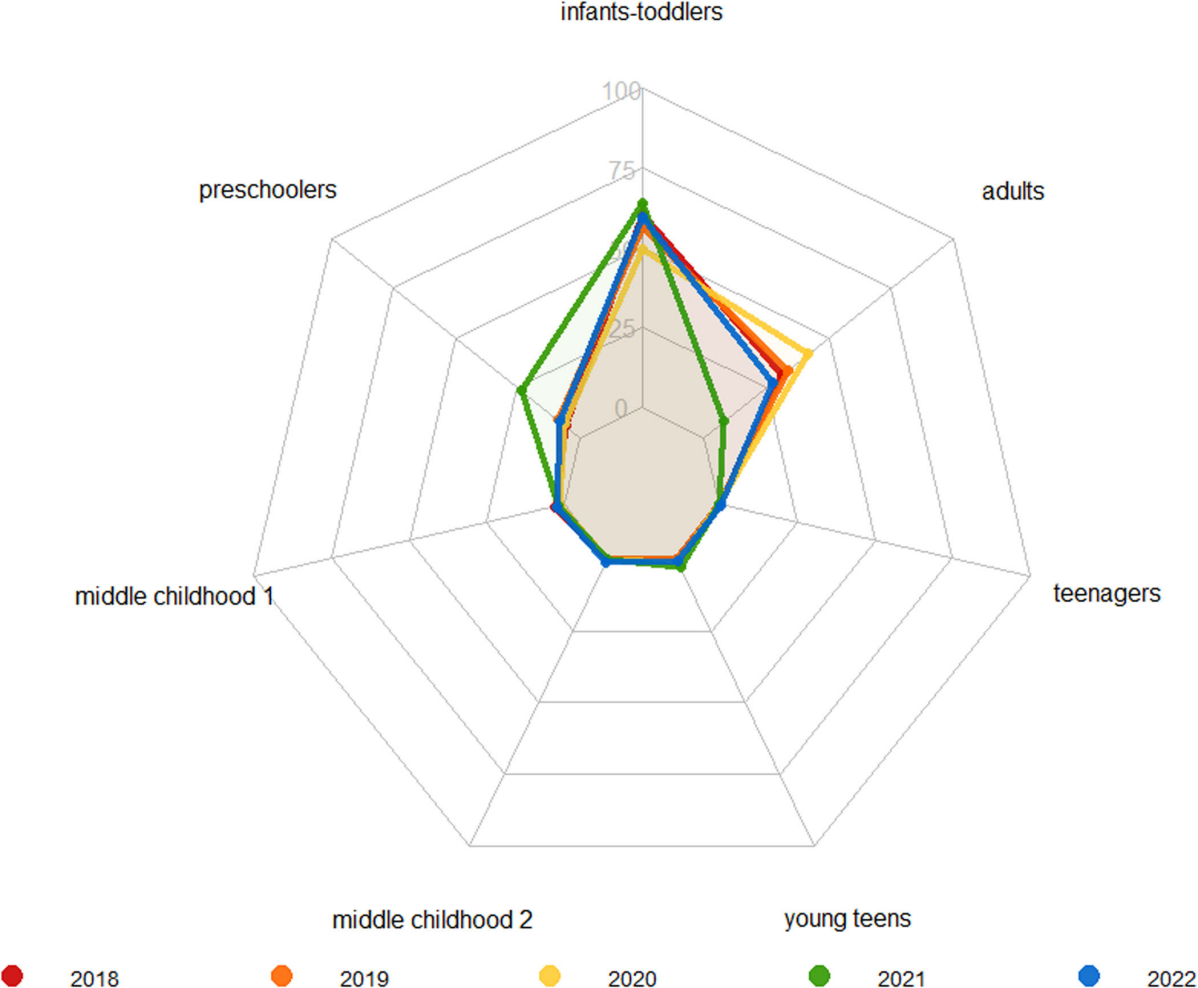
RSV positivity rate between 2018 and 2022. Spider chart shows the percentage of RSV positive samples between 2018 and 2020. Our cohort was divided in paediatric population and adults (>18 years). Paediatric population was then stratified in infants‐toddlers (aged 0–2 years), preschoolers (aged 3–5 years), middle childhood people (group 1: aged 6–8 and group 2: 9–11 years), young teens (aged 12–15) and teenagers (aged 16–18).

Additionally, in 2021 the positivity rate in adults decreased to 7.8% from 31% and 33.8% in pre pandemic years, and 41.8% in 2020, it then increased again to 27.2% in 2022. These findings suggest a possible shift in the virus epidemiology likely due to different age of the infected individuals and/or environment in which it can be transmitted.^1^ Negative tests were equally distributed in the period 2018–2022 in this groups besides in 2020, corresponding to the beginning of the pandemic, when the increase of tests may be related to SARS‐CoV‐2 differential diagnosis (Figure 2B).

RVs, known to be highly resistant to environmental factors, were not less affected by NPIs.^11^ We estimated a positivity rate of 39.2% of 79 samples in 2018, 31.8% of 579 in 2019, 5.1% of 2752 in 2020, 19.3% of 1225 in 2021 and 17.5% of 1846 in 2022. Interestingly, no differences were found about the spread of other respiratory viruses.

In conclusion, our data highlight that pre‐ and postpandemic respiratory virus circulation remains substantially unchanged, besides slight variations in seasonality and NPIs impact.

We believe that further in‐depth studies will be needed to unveil the incidence and spread of respiratory viruses which may have been widely underestimated in both the prepandemic and pandemic period in which testing was not as prevalent as it is now.^12^ Epidemiological studies together with evolutionary genomic observations will shed light on the landscape of respiratory virus infections and help to clarify whether currently observed trends are associated to radical changes in the diagnostic approach and a wider availability of testing in the postpandemic world.

## AUTHOR CONTRIBUTIONS

**Flavio De Maio**: Conceptualization; data curation; formal analysis; investigation; methodology; software; supervision; validation; visualization; writing—original draft; writing—review & editing. **Barbara Fiori**: Data curation; software; validation; visualization. **Delia Mercedes Bianco**: Formal analysis; visualization; writing—original draft; writing—review & editing. **Maurizio Sanguinetti**: Resources; supervision; visualization; writing—review & editing. **Michela Sali**: Data curation; supervision; visualization; writing—review & editing.
